# P-603. Potential Public Health Impact of Respiratory Syncytial Virus (RSV) Vaccines for Prevention of RSV Among Older Adults in the United States

**DOI:** 10.1093/ofid/ofae631.801

**Published:** 2025-01-29

**Authors:** Ahuva Averin, Reiko Sato, Erica Chilson, Mark Atwood, Erin Quinn

**Affiliations:** Avalere Health, Boston, Massachusetts; Pfizer, Inc., Collegeville, Pennsylvania; Pfizer, 500 Arcola Road, Pennsylvania; Avalere Health, Boston, Massachusetts; Avalere Health, Boston, Massachusetts

## Abstract

**Background:**

Two United States (US) Food and Drug Administration-approved vaccines are recommended by the Advisory Committee for Immunization Practices for prevention of lower respiratory tract disease (LRTD) due to respiratory syncytial virus (RSV) among US adults aged ≥ 60 years and another vaccine for adults is under regulatory review. We evaluated the potential public health impact of vaccines for prevention of RSV-LRTI in older adults.

**Figure:**
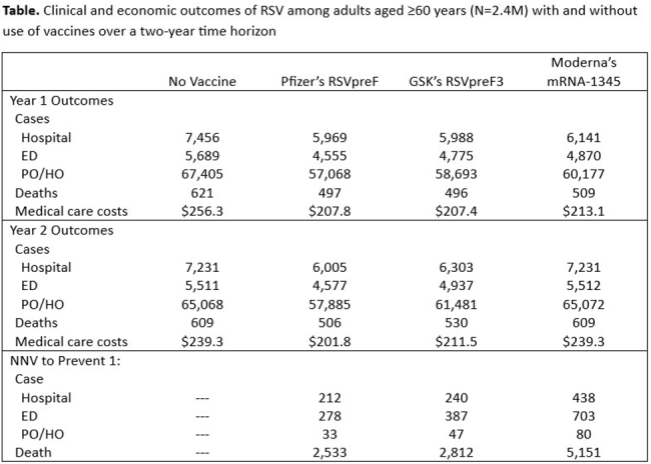

**Methods:**

A cohort model was employed to depict clinical outcomes and economic costs of RSV-LRTD over two years with use of alternative vaccines (ie, Pfizer’s RSVpreF, GSK’s RSVpreF3, Moderna’s mRNA-1345 respectively, vs no intervention) among a cohort of 2.4M adults aged ≥ 60 years in a hypothetical US health plan with 10M beneficiaries. Clinical outcomes were projected (monthly) based on age, comorbidity profile (ie, with vs without chronic or immunocompromising medical conditions), RSV-LRTI disease rates (requiring hospitalization [H], emergency department [ED], or physician office/hospital outpatient care [PO/HO]), calendar month, case fatality rates (RSV-H only), and vaccination status (23.6% uptake in October of model year 1 only). Initial vaccine effectiveness (VE) and waning of VE was derived from published or presented Phase III clinical trial data. Economic costs were generated based on cases and corresponding unit costs.

**Results:**

Without use of RSV vaccine, 14,687 hospitalizations, 11,201 ED encounters, 132,472 PO/HO visits, and 1,230 RSV-related deaths are projected to occur over two years. Pfizer’s RSVpreF would result in a 22,301-case reduction (RSV-H: 2,713; RSV-ED: 2,069; RSV-PO/HO: 17,519) and 227 fewer deaths; total medical care costs would be lower by $86.0M (RSV-H: $64.2M; RSV-ED: $7.4M; RSV-PO/HO: $14.4M) (Table). GSK’s RSVpreF3 would reduce cases by 16,183, deaths by 205, and medical care costs by $76.8M. Moderna’s mRNA-1345 would reduce cases by 9,357, deaths by 112, and medical care costs by $43.2M (based on single-season efficacy data).

**Conclusion:**

RSV vaccines have the potential to substantially reduce the clinical and economic burden of RSV-LRTI in older adults. Of the three vaccines, Pfizer’s RSVpreF would provide the greatest benefit due to its high 2-year efficacy.

**Disclosures:**

**Ahuva Averin, MPP**, Pfizer Inc: Advisor/Consultant **Reiko Sato, PhD**, Pfizer Inc: employee|Pfizer Inc: Stocks/Bonds (Private Company) **Erica Chilson, PharmD**, Pfizer Inc: Employee|Pfizer Inc: Stocks/Bonds (Public Company) **Mark Atwood, MS**, Pfizer Inc: Advisor/Consultant **Erin Quinn, BS**, Pfizer Inc: Advisor/Consultant

